# Using the ISM-ANP-SD combination model to explore the mechanism and intervention strategies of influencing factors of coal mine safety system

**DOI:** 10.3389/fpubh.2022.1053298

**Published:** 2022-11-23

**Authors:** Xue Yang, Qiyu Xing, Kang Tian, Chen Liu, Juan Yang

**Affiliations:** ^1^School of Management and Economics, North China University of Water Resources and Electric Power, Zhengzhou, China; ^2^College of Information and Management Science, Henan Agricultural University, Zhengzhou, China

**Keywords:** coal mine safety system, human-machine safety collaboration, interpretive structural model, network analytic hierarchy process, system dynamics

## Abstract

**Background:**

With the intelligent construction of coal mines, the number of coal mine accidents is gradually decreasing, but the complexity of accidents is increasing. Understanding the interaction mechanism among the influencing factors of the coal mine safety system is an essential part of improving and enhancing the safety of the coal mine system.

**Methods:**

The descriptive, structural model-network hierarchical analysis (ISM-ANP) was used to explore the interaction between the factors influencing the coal mine safety system and determine each factor's importance. A system dynamics simulation model was constructed to clarify the mechanism of each factor's effect on the safety system.

**Results:**

The results show that Individual miners' factors directly influence coal mine system safety, organizational management factors, and group factors indirectly influence system safety and play the role of macro regulation. The intelligent system is the most profound factor influencing system safety. There are apparent differences in the influence of different subsystems on system safety, with organizational management having the most significant influence on system safety, followed by individual miners and group factors, and intelligent system factors and external environmental factors having a more negligible influence on system safety.

**Conclusion:**

There is a complex interaction between the factors affecting the safety of the coal mine system, and there are apparent differences in the influence of different subsystems on the safety level of the coal mine system. This study puts forward the intervention strategy to improve the safety of the coal mine system, which provides theoretical support and method guidance for preventing coal mine accidents and improving the safety level of the coal mine system.

## Introduction

The rapid development of the mining industry has contributed significantly to China's national economy and social development (1). It is essential to ensure the safety and sustainability of mining operations (2). Caravalho believes sustainable mining depends on better environmental protection, long-term management of natural resources, equitable socio-economic impact, and improved safety of mining activities (3). Among them, ensuring the safety of mining activities is seen as a fundamental determinant of sustainable mining (2, 4). With the continuous improvement of automation and information level in the coal industry, coal enterprises attach great importance to sustainable mining. They have made beneficial attempts and explorations to improve the sustainability of the mining industry and coal mine safety through intelligent construction (5). The achievement of intelligent construction of coal mines at this stage is mainly reflected in the realization of informatization in primary links such as coal mine development and design, geological guarantee, production, and safety. The reduction of personnel, the improvement of efficiency, and the fewer people operating also symbolize the completion of intelligent construction on the coal mining and excavation surfaces, respectively (6). Although coal mine fatalities and accidents are gradually decreasing, the complexity of coal mine accidents is increasing (7). Finding out the reasons for the complex changes in coal mine accidents and systematically analyzing coal mine accidents is an urgent problem to be solved in coal mine safety management under the intelligent construction of coal mines.

The coal mine safety system is a dangerous and dynamic complex system composed of coal mine safety-related elements such as natural conditions, equipment, management systems, and several active subjects (8). System safety is an essential basis for the safe production and operation of coal mines, maintaining daily stability, safety, and economy. With the intelligent construction of coal mining enterprises, the original production equipment or systems have been gradually upgraded and replaced. The application of new technologies allows more factors (such as automation trust and automation dependence) to be introduced into the security system, increasing the complexity inside the system. At this point, if any part of the coal mine safety system fails, it creates a safety hazard and may lead to a coal mine accident. Therefore, it is necessary for coal mining enterprises to shift the focus of safety management to the safety management of coal mine systems and to find out the factors that cause complex changes in coal mine accidents. Researchers need to understand the complexity, evolution law, and operation mechanism of the coal mine safety system by exploring the interaction relationship between the factors of the coal mine safety system and the interaction mechanism between the various subsystems in the coal mine safety system. Coal mine safety accidents can be effectively prevented only with the joint efforts of many parties.

Subsystems and their attributes determine the safety of a coal mine system, and the behavior interaction between subsystems is complex and changeable. The occurrence of an accident is not the result of a single factor but the interaction of many factors (9). Therefore, it is essential for mine safety risk prevention and system safety to deeply understand the causative factors and action mechanisms of accidents (10). Scholars tend to use Decision Making Trial and Evaluation Laboratory and Interpretative Structural Modeling Method (DEMATEL-ISM) (11), data mining (association-rule and decision tree) (12), Structural Equation Model (SEM) (13), Bayesian Networks (BN) (14) and other methods to study the factors influencing coal mine safety and action mechanism. However, previous studies mainly discussed the influence of a single factor and the causal relationship between factors on the accident. The internal interaction between factors and the hierarchy of factors have yet to be deeply studied. There needs to be a more systematic and dynamic analysis of the cause of the accident. ISM can divide a complex system with complex structure and fuzzy logic into several related subsystems, and construct a multi-layer hierarchical structure model, thereby dividing the influence paths and hierarchical structures among factors (15). The ISM has many applications in exploring the hierarchical relationship and correlation between factors (16, 17). It is difficult for the ISM to reflect the relative importance of each element in the entire system. At the same time, Analytical Network Process (ANP) is a multi-criterion weighted decision-making method that can reflect the mutual influence between indicators. ANP can perform a limited ordering of itemsets, more accurately describe the network structure among factors, and complement the computational results of ISM with quantitative analysis (18). ISM-ANP can effectively reflect the influence degree and path of various factors on the coal mine safety system. The current coal mine safety management system has the characteristics of non-linearity and a feedback loop. It is a complex and dynamic man-machine-environment-management system with intertwined effects of multiple factors (9). The dynamic evolution analysis can better reflect its complex and dynamic characteristics. System dynamics (SD) can combine quantitative and qualitative analysis with studying the interaction of various factors in complex systems through model simulation and has a wide range of applications in risk assessment and safety management (19, 20).

Advances in technology have made systems more complex, especially the complexity of the interactions between factors within the system. More understanding of the causal path of complex systems and the dynamic evolution law of system safety may increase the system risk and safety level of the coal mine safety management system. Therefore, based on analyzing the influencing factors of the coal mine safety management system under the background of coal mine intelligent construction, this study proposes a system safety management method combining ISM-ANP-SD. By constructing the ISM-ANP-SD model, we explored the causal path of coal mine safety accidents and the dynamic evolution law of coal mine system safety. The research results can provide the scientific basis for accident prevention and improvement and provide a reference for the study of coal mine system safety management.

## Analysis of coal mine safety system

### Analysis of influencing factors of the coal mine safety system

Identifying influencing factors of the coal mine safety system is also the process of comprehensively identifying coal mine safety risks. Any factors influencing safety and health related to people, Intelligent systems, and the environment should be considered (21). Scholars have researched the factors influencing the coal mine safety system. Liu et al. (22) analyzed the coal mine safety accidents from the external environment, organizational factors, poor leadership, preconditions for unsafe behavior, and unsafe behavior. Fa et al. (23) divides the factors that restrict the safety production of coal mines into seven aspects: unsafe behavior, unsafe preconditions, unsafe supervision, organizational influence, external influence, mechanical equipment factors, and physical environment factors. Jiskani used fuzzy synthetic evaluation to evaluate 41 risk factors influencing sustainable mining in Pakistan. They classified the risks into 8 categories: Economic and financial, Environmental, Health and safety, Natural and external, Operational and technical, Organizational and managerial, Political and legal, and Socio-cultural (1). Based on the systematic theory of human, machine, environment, and management, Bai and Xu constructed the classification model of coal mine safety evaluation, constructed 14 evaluation index systems from four aspects of human, machine, management, and environment, and used BP neural network to evaluate coal mine safety (24). Ma established 30 evaluation index systems from five aspects: environmental disaster, safety management, facility performance, behavior monitoring, and emergency rescue. AHP, Entropy method, and multi-granularity non-equilibrium semantic treatment method are used to calculate the index's weight, and the suggestion of coal mine safety management is put forward (25). To sum up, scholars' studies on influencing factors of coal mine safety mostly construct index systems from individual factors (26), organizational factors (11, 22), management factors (27, 28), machinery and equipment (29, 30), and environmental factors.

**Figure 1:**
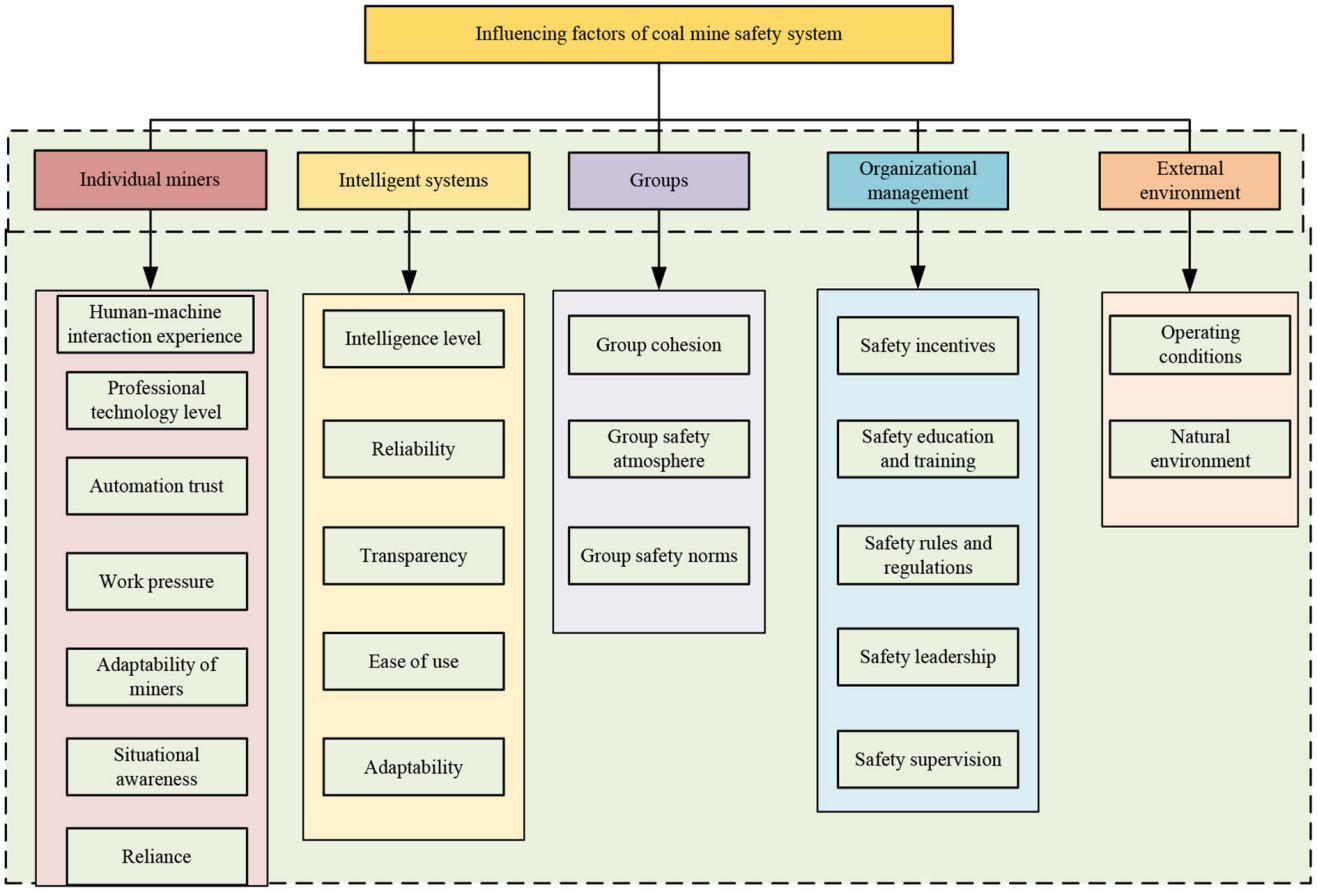
Influencing factors of coal mine safety system.

Under the intelligent construction of coal mines, much new intelligent equipment, sensors, and automatic controllers have appeared in the production process, and the stability and reliability of the equipment have been greatly improved (5). The relationship between miners and coal mine machinery and equipment has changed from the traditional “people-oriented, machine-assisted” to the current “human-machine cooperation (31).” Therefore, under the intelligent construction of coal mines, the interaction process of the “human-machine-management-environment” should be considered more in the research on the influencing factors of the coal mine safety system. According to the actual situation of the intelligent construction of coal mines in China and related research results, this study mainly divides the influencing factors of coal mine safety system into five aspects: individual miners, intelligent systems, organizational management, groups, and environment, and determines 22 influencing factors, and constructed a map of the influencing factors of the coal mine safety system, as shown in Figure 1.

### Theoretical model building

Three-dimensional interactive decision-making believes that human behavior, the internal factors of actors, and their environment are independent, continuous, and dynamic interactions. The mode of action between the three is not constant and will show different modes of influence according to specific situations (32). The ternary interactive determinism links people's behavior, the actor's internal factors, and the actor's environment and builds the interaction model of the three. Among them, the internal factors of the actor mainly refer to the individual's psychological functions, such as cognition, emotion, belief, expectation, and attitude. External environmental factors include physical and social factors such as work resources, organizations, and leaders. Behavior mainly refers to the individual's choice of action.

The “stimulus-response” theory originated from behaviorist psychology. Jacoby (33) added individual cognition to the “stimulus-response” theory and proposed the “stimulus-organism-response” theory (S-O-R theory). The S-O-R theory holds no direct interaction between external environmental stimuli and individual responses. An individual is an organism with rich emotions and cognitive activities and has subjective initiative. Therefore, the individual is not simply a passive response to the stimulus but produces a specific psychological activity, which influences the individual to make an active choice.

**Figure 2:**
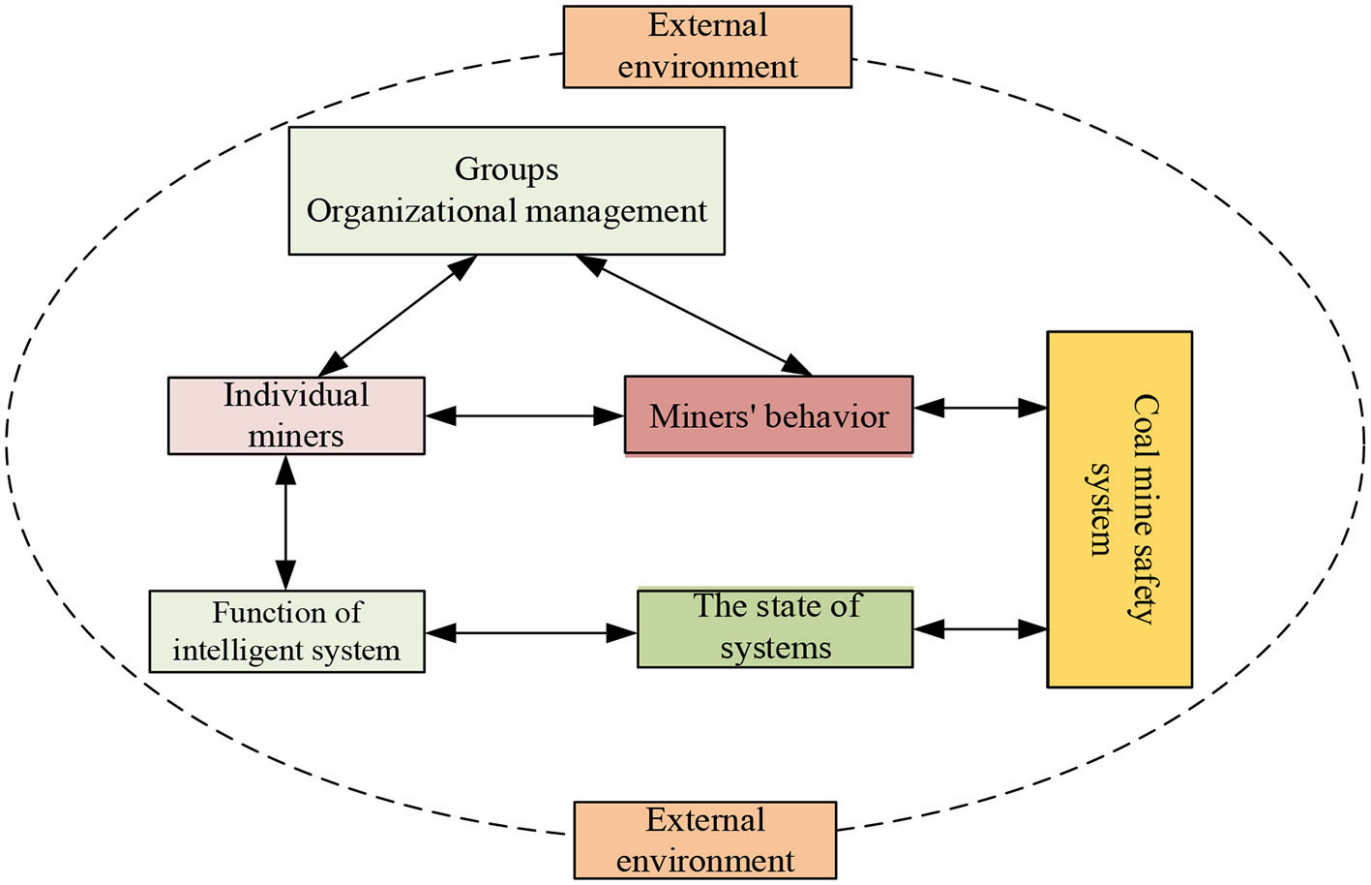
Theoretical framework of coal mine safety system.

The coal mine safety system consists of five subsystems: individual miners, intelligent systems, organizational factors, external environment, and management factors. The coal mine safety system is a human-centered man-machine matching system with a feedback process. The user's demand for the system leads to the functional interaction and elastic interaction of each subsystem according to the design requirements; that is, the regular operation of each subsystem depends on the normal operation of the functions of other subsystems associated with it. Combined with ternary interactive determinism, “stimulus-body-response” theory, and according to the actual production situation of the coal mining industry, a conceptual model of the coal mine safety system is constructed, as shown in Figure 2.

## Research methods

In this paper, the improved ISM based on DEMATEL is used to analyze the hierarchical structure and internal interaction among factors influencing the coal mine safety system from a qualitative point of view. ISM can simplify and regularize the complex and chaotic system by building a multi-level hierarchical structure model to clarify the system's hierarchical structure and internal interaction relationship (15). This method stratifies the influencing factors in the system, which can better solve the problem that there are many influencing factors in the system, and the correlation between the factors is relatively complex and can analyze the core factors in the system and the correlation between the influencing factors. However, the ISM method only considers binary relationships and unidirectional effects between factors when considering the relationships of factors. The direct influence matrix constructed by the DEMATEL method can more accurately describe the strength of the interactions and influence relationships between factors (34). Therefore, when considering the initial relationship between system elements, the improved ISM is based on DEMATEL. The improved ISM can be used to determine the strength of the influence relationship between factors by constructing the direct influence matrix, which has better applicability and operability than the adjacency matrix built by traditional ISM. In addition, when calculating the reachability matrix, the improved ISM based on DEMATEL can introduce a threshold to screen the action paths and retain the critical path. The ANP method analyzes the relative importance of factors according to the influence relationship of factors in the system, which can quantitatively supplement the research results of ISM. Moreover, ISM and ANP methods are based on the influence relationship between factors to analyze the system and have the same application conditions. Many factors influence the coal mine safety system, and their interaction forms a complex network of influence relations. Based on this characteristic, ISM-ANP is suitable for analyzing the coal mine safety system's action path and key influencing factors.

**Figure 3:**
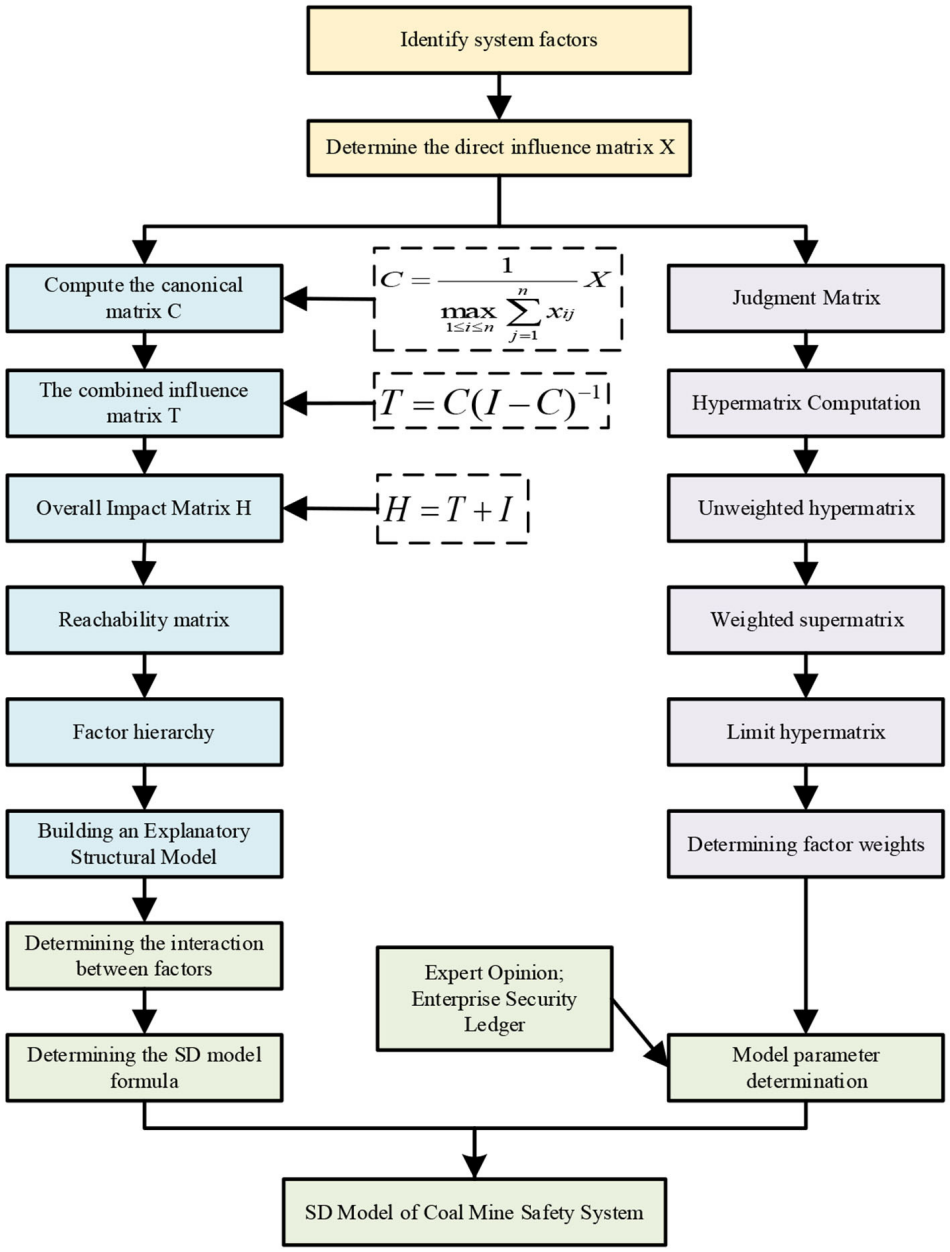
Flow chart of ISM-ANP-SD model of coal mine safety system.

System dynamics is based on information theory, cybernetics, and system theory and uses computer technology to simulate and analyze the dynamic and complex causal relationships of various elements and to understand complex systems as a whole. System dynamics is a quantitative research method to study high-order, non-linear, multivariable complex systems. The influence of various factors in the coal mine safety system is complex, dynamic, and implicit. It is necessary to observe the evolution process of safety in the system from a dynamic perspective. The method of system dynamics is suitable for revealing this dynamic evolution law. The specific method steps of the ISM-ANP-SD model of the coal mine safety system constructed in this study are shown in Figure 3.

## Results and discussion

### Model building

#### ISM-ANP model construction

Determine the direct impact matrix. Ten experts were invited to rate the correlation of 22 factors influencing the mine safety system. The invited three intelligent, fully mechanized face technicians have been involved in the intelligent transformation of coal mine enterprises for a long time. They have a complete understanding of the technology adopted in the intelligent construction of coal mines and the situation of the coal mining site and have rich theoretical and practical experience. Four front-line miners have been engaged in coal mining for a long time and fully understand the factors influencing the safety of the coal mine system. Three professors in the field of coal mine safety have been actively involved in the research of coal mine safety management in the past 10 years and have rich experience in coal mine safety management. The 10 experts invited by this study have strong theoretical and practical experience, which can ensure the reliability and validity of the data. The number 0–4 is used to indicate the degree of influence between factors, where “0” means no influence, “1” slight influence, “2” general influence, “3” strong influence, and “4” strong influence. The scoring value of each expert is averaged to obtain the direct influence matrix of each influencing factor.

**Figure 4:**
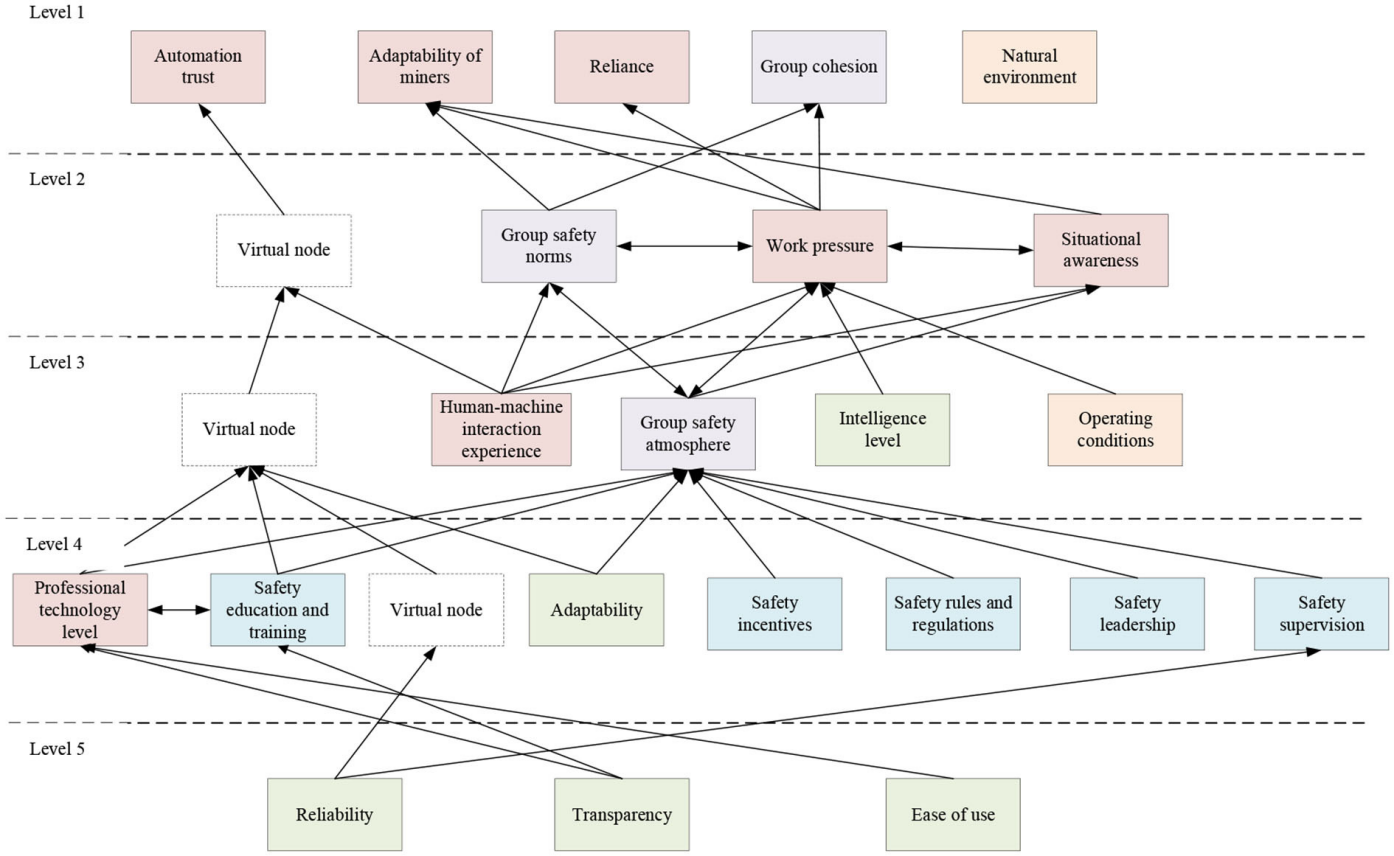
Interpretation structure model of coal mine safety system.

MATLAB software calculates the adjacency matrix, and the reachable matrix is obtained. Determine the reachable set *R*_*i*_ and the antecedent set *S*_*i*_ of the reachability matrix. The satisfying factors *R*_*i*_ = *R*_*i*_∩*S*_*i*_(*i* = 1, 2, ..., 22) are the factors of the first level of the system, deleting the elements of the first level, calculating according to the formula, analyzing the system layer by layer, and obtaining the hierarchical table of factors. The explanatory structure model of the coal mine safety system constructed according to the reachability matrix and the hierarchical table is shown in Figure 4.

**Figure 5:**
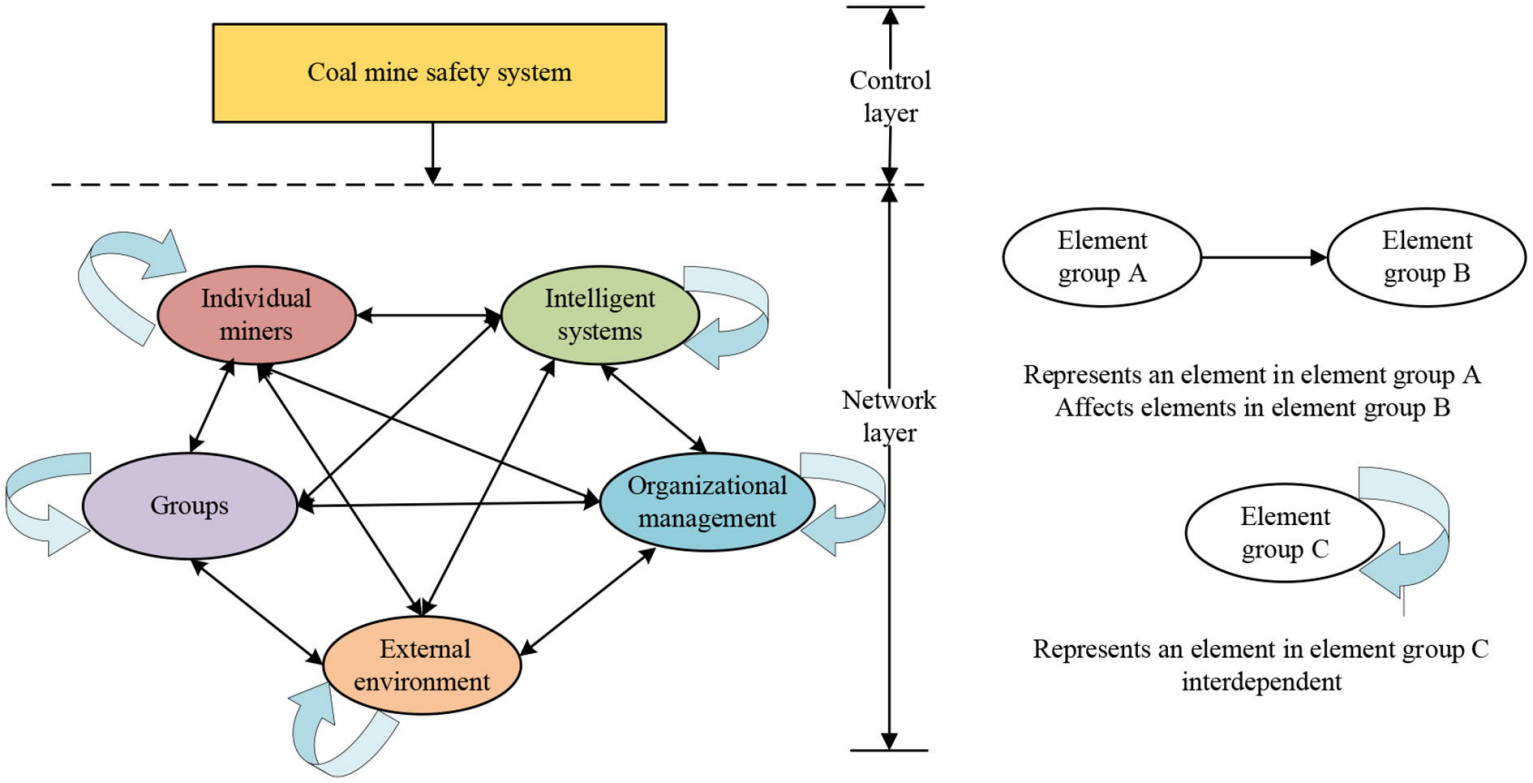
Network structure model of coal mine safety.

Calculate the judgment matrix. If the direct influence matrix shows an influence relationship between the two factors, the value of the corresponding judgment matrix is 1. If there is no influencing relationship between the two factors, the corresponding judgment matrix value is 0. According to the judgment matrix results, input the Super Decision software to obtain the network structure model of ANP, as shown in Figure 5, and construct 5 cluster judgment matrices and 80 node judgment matrices. Ten experts involved in the ISM were invited to score the judgment matrix using the “1–9 scale method”, and the Super Decision software was used to calculate the weight of each factor. Each calculation result passed the matrix consistency test. The weight calculation results of the ANP model are shown in Table 1.

**Table 1:** ANP weight calculation results.

**First-level indicator**	**First-level indicator weight**	**Secondary indicators**	**Global weight**	**Local weights**
Individual miners	0.27	Human-machine interaction experience	0.017	0.06
		Professional technology level	0.041	0.16
		Automation trust	0.035	0.13
		Work pressure	0.04	0.15
		Adaptability of miners	0.053	0.2
		Situational awareness	0.051	0.19
		Reliance	0.028	0.11
Intelligent systems	0.11	Intelligence level	0.003	0.02
		Reliability	0.042	0.37
		Transparency	0.022	0.19
		Ease of use	0.028	0.25
		Adaptability	0.019	0.17
Groups	0.21	Group cohesion	0.079	0.39
		Group safety atmosphere	0.077	0.38
		Group safety norms	0.049	0.24
Organizational management	0.37	Safety incentives	0.079	0.22
		Safety education and training	0.083	0.23
		Safety rules and regulations	0.052	0.14
		Safety leadership	0.09	0.25
		Safety supervision	0.062	0.17
External environment	0.05	Operating conditions	0.035	0.7
		Natural environment	0.015	0.3

#### Analysis and discussion of the results of the ISM-ANP model

There is a complex relationship between the influencing factors in the coal mine safety system. In the ISM, the first-level factors of automatic trust, miners' adaptability, dependence, organizational cohesion, and natural environment are the most direct factors that influence the safety of the coal mine system. Interventions on these factors can directly and effectively promote the safety level of the coal mine system. The factors at the second, third and fourth levels are important factors that influence the coal mine safety system. Most of these are related to management and organizational factors. Although the factors at the second, third and fourth levels have no direct impact on the safety of the coal mine system, they can influence the safety of the coal mine system by influencing the state of the miners. The fifth-level influencing factors reliability, transparency, and ease of use are the most profound influencing factors. These three factors are all related to the intelligent system and will not directly lead to coal mine safety accidents. However, it directly impacts miners' behavior and the level of organization and management and is the most fundamental cause of coal mine safety accidents.

Through the ANP calculation results, it can be seen that the ranking of the impact on the coal mine safety system in the first-level indicators is: organizational management, individual miners, groups, intelligent systems, and environment. Combined with the results of ISM, it can be seen that organizational management factors, individual miners, and group factors have a more significant impact on system safety, and individual miners directly influence the coal mine safety system. Organizational and group factors indirectly influence the coal mine safety system and play a role in macro-control. Appropriate intervention on organizational management and group factors can effectively improve the safety behavior of miners. The impact of intelligent systems and environmental factors on system security is tiny. However, the intelligent system's good working performance and good working environment ensure the entire system's security.

#### System dynamics model construction

**Figure 6:**
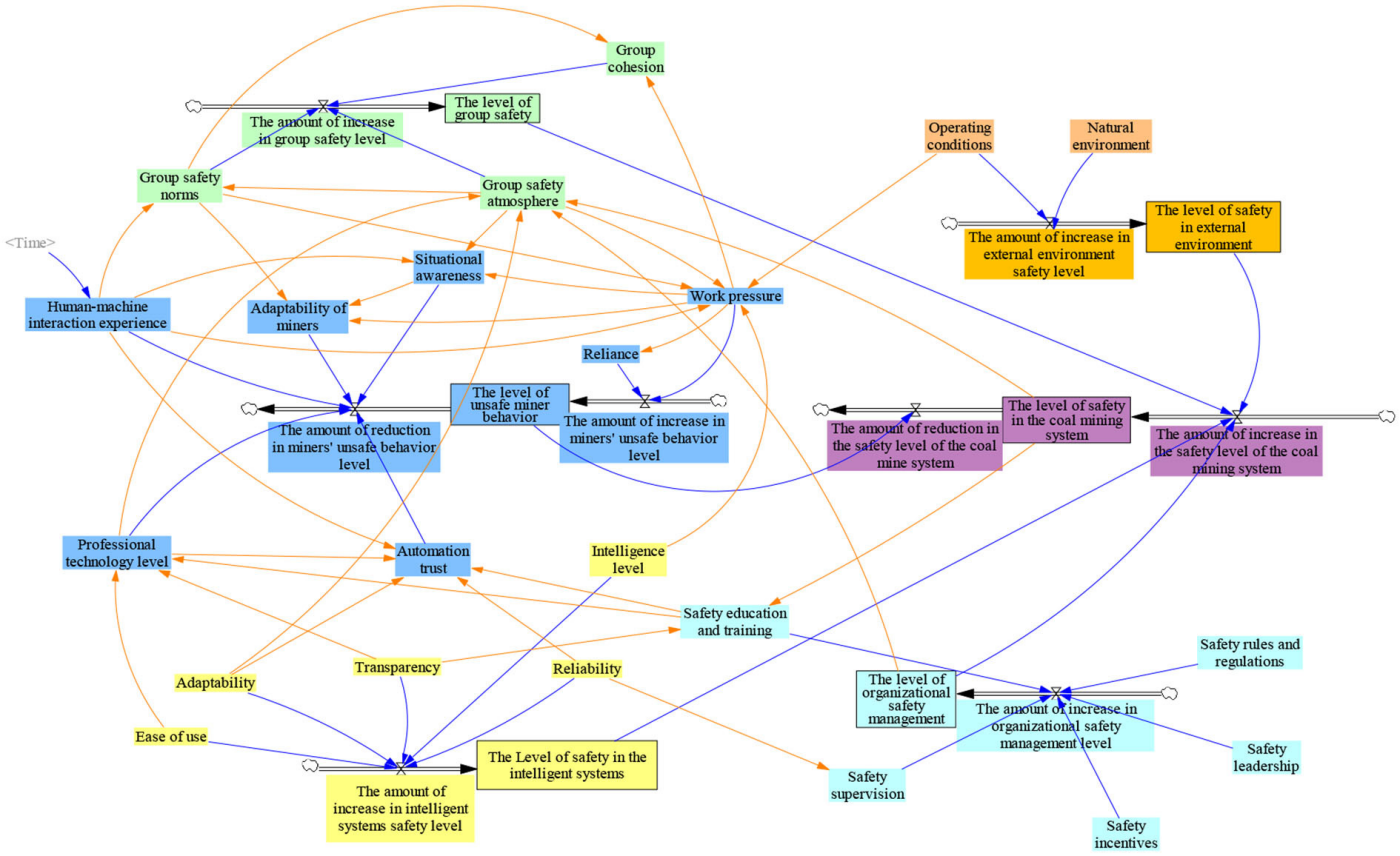
Stock-flow diagram of coal mine safety system.

The principles of purpose, applicability, validity, and simplicity must be followed when building a system dynamics model. Each influencing factor finally influences the safety of the coal mine system by influencing miners' unsafe behavior subsystem, intelligent system subsystem, organization management subsystem, group subsystem, and environmental subsystem. According to the interaction relationship between the factors obtained by ISM, Vensim PLE software is used to draw the safety stock-flow map of the coal mine system, as shown in Figure 6. Set the model Initial time = 0, Final time = 24, Units = Month. The simulated data comes from Pingmei No. 4 Mine, owned by China Pingmei Shenma Energy and Chemical Group Co., Ltd. Field research was conducted in Pingmei No. 4 Mine to obtain the operation ledger data from May to August 2021. Interviews were conducted with the intelligent coal face's management personnel, technical personnel, and front-line miners. The initial values of state and auxiliary variables in the SD model were determined by combining the coal mine safety system operation ledger data and interview data. The influence coefficient between variables is mainly determined based on the ANP calculation results and expert interviews.

### Model simulation results and analysis

#### Analysis of initial simulation results

**Figure 7:**
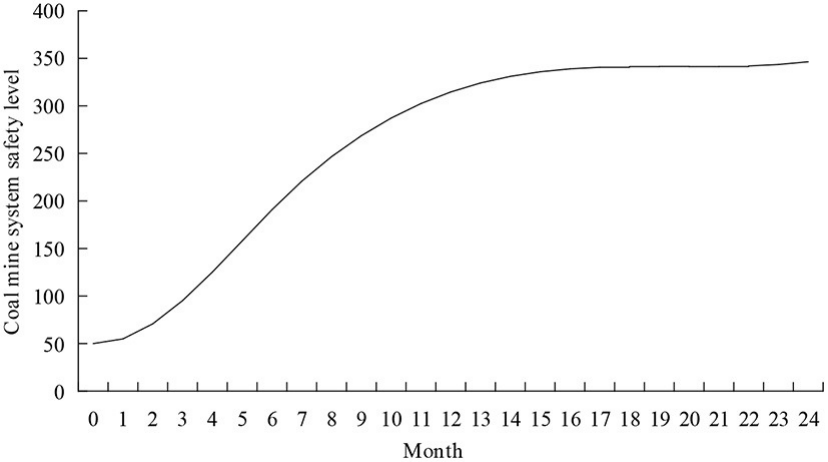
Initial simulation results of coal mine system safety level.

Models were tested for construct validity, dimensional consistency, and historical values before model simulation and analysis. The model's structural validity test ensures the rationality of the logical relationship of the model. The relationship between the variables in Figure 6 is determined based on the interpretation of the structural model combined with the actual situation of coal mine production, ensuring the model's rationality. The dimensional consistency check ensures whether the dimensions used by the equations and parameters in the model are appropriate. The model is checked for dimensional consistency using the model checking function in the Vensim software and passed. The historical value test of the model verifies the safety value of the coal mine system for 1–6 months. The error rate between the simulated value and the original value is 3.9% < 5%, indicating that the model has a reasonable degree of fitting and that the model in this study is valid. The initial simulation results of coal mine system safety are shown in Figure 7.

According to Figure 7, the change in coal mine system safety can be divided into three stages. (1) During the rapid rise period from 0 to June, the safety level of the coal mine system increased from 50 to 190.757: On the one hand, in the interaction process between miners and intelligent systems, the experience of miners is rapidly accumulated, the adaptability is gradually increased, and the level of automation trust and situational awareness is also improved with human-machine interaction. At this time, miners' work pressure level and dependence on automation are low, and the unsafe behavior of miners is low. On the other hand, due to the high level of safety supervision and safety education and training for miners by managers in the early stage of human-machine interaction, the safety level of the coal mine system has been increased. (2) During the rising period from June to December, the safety level of the coal mine system increased from 190.757 to 314.636: Miners' work pressure and automation dependence increased unsafe behavior in this stage. The gradual familiarization of team members will improve team cohesion and a safe atmosphere. This can effectively alleviate the increase in miners' unsafe behavior caused by work pressure and automation dependence. (3) During the slow-rising period from December to 24, the safety level of the coal mine system increased from 314.636 to 346.374: At this stage, the level of unsafe behavior of miners, the level of environmental safety, and the level of organizational management are gradually stabilized, the group safety norms within the group are gradually formed, and the group cohesion and group safety atmosphere grow slowly. Therefore, the safety level of the coal mine system grows slowly in this stage.

#### Analysis and discussion on simulation results of intervention strategy

Individual miners, organization management, intelligent systems, groups, and the environment have operation rules. Each subsystem interacts with the other, and different subsystems have different effects on the behavior of other subsystems and the safety of the coal mine system. The factors of each subsystem are intervened. The influencing mechanism of each factor on the safety of the coal mine system is discussed to provide policy suggestions for improving the safety level of the coal mine system.

#### The intervention in the individual subsystem of miners

**Figure 8:**
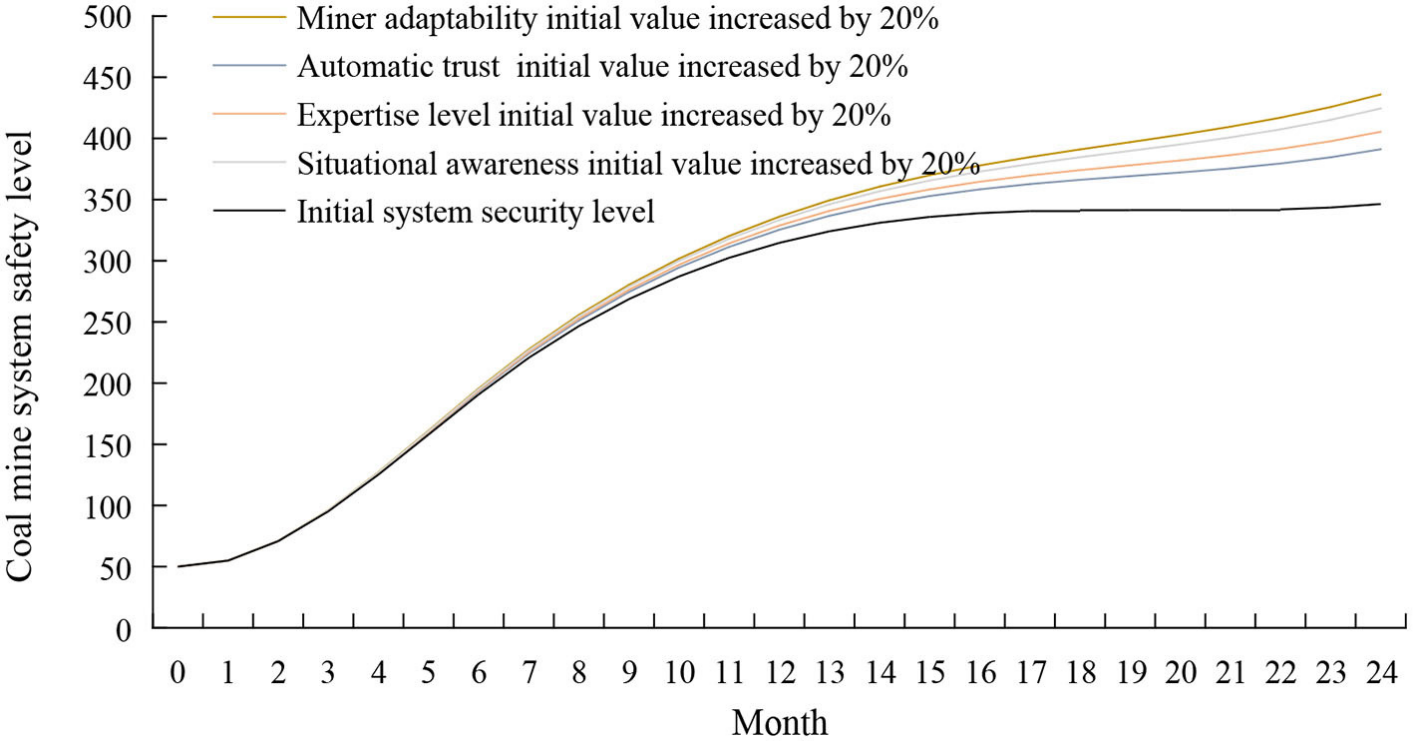
Simulation results of coal mine system safety after the intervention of miners' individual subsystems.

In the individual subsystem of miners, human-machine interaction experience is a time-related variable, and human intervention factors have little impact on it. Work pressure will lead to an increase in the level of miners' unsafe behavior, which negatively influences system security. In order to improve the safety level of the coal mine system, the control variable method is used to increase the initial values of professional technology level, automation trust, situational awareness, and miner adaptability by 20% while keeping other variables unchanged. The simulation results are shown in Figure 8. It can be seen from Figure 8 that after the intervention strategy is adopted, the safety state of the coal mine system is improved compared with the initial state, and the effect is more evident at 7–24 months. The effect on the safety of the coal mine system is from large to small: adaptability of miners, situational awareness, professional technical level, and automation trust.

The tasks and responsibilities performed by miners have undergone significant changes. The complexity of the intelligent system requires miners to have higher adaptability to identify and take corresponding security measures on time in the face of crises (35). The adaptability of miners is the primary ability of employees under the intelligent construction of coal mines. The intelligent construction of coal mines requires miners to have the ability to adapt to pressures, dangers, and emergencies (36). To improve the adaptability of miners, miners must have continuous learning ability and actively understand the principles and methods of equipment. In addition, managers should strengthen risk identification and cognitive training for miners and improve miners' adaptability to dangerous and unexpected situations.

Situational awareness is the perception and understanding of entities in the environment and the prediction of entity states (37). The importance of situational awareness to the security of complex systems has long been demonstrated (38, 39). It can be seen from the model analysis that human-machine interaction experience and group safety atmosphere have a positive impact on miners' situational awareness. With the increase of human-machine interaction experience and the improvement of group security atmosphere, miners' situational awareness level will increase; work pressure may cause miners to respond slowly and have no apparent sense of system risk. Managers can improve miners' situational awareness by improving the group's safety atmosphere, using miners with experience in human-machine interaction, and timely dredging miners' work pressure.

Relevant knowledge and technical skills are critical to miners' safety awareness (40). Improvements in intelligent coal mine equipment should be accompanied by updating miners' relevant knowledge and technical skills. Professional knowledge and technical skills help miners understand the operating methods and operation level of intelligent systems and reduce the occurrence of unsafe miner behavior. There are two ways to improve miners' expertise: improving the HMI design and enhancing safety education and training for miners. In the design of the HMI, ease of use and transparency should be improved to provide operators with a reliable reference for decision-making (41). Strengthening the education of miners' operating knowledge and operating principles, as well as the training of operating skills, pattern recognition, and pattern matching training, can also effectively improve the professional technical level of miners.

Automated trust influences miners' use of intelligent systems (42). Over-trust or lack of trust will lead to over-supervision or under-supervision of intelligent systems by miners (43), making miners react poorly in critical events and leading to safety accidents (44). Therefore, an appropriate level of trust is essential: operators must understand the capabilities of intelligent systems and adequately monitor them as they approach the limits of their capabilities (45). Maintaining an appropriate level of trust among miners begins with training miners with a clear and detailed introduction to the functions and operation of the intelligent system, explaining the system's limitations, and improving the miners' level of expertise. Second, improve the human-machine interface design of the intelligent system to improve the system's explanation capability so that when the system malfunctions, the system can be explained verbally or visually to the operator promptly to facilitate the operator's understanding of the intent and actions of the intelligent system (46). Finally, miners are encouraged to think positively and try to resolve their distrust of the intelligent system due to personal reasons.

#### The intervention in the organizational management subsystem

**Figure 9:**
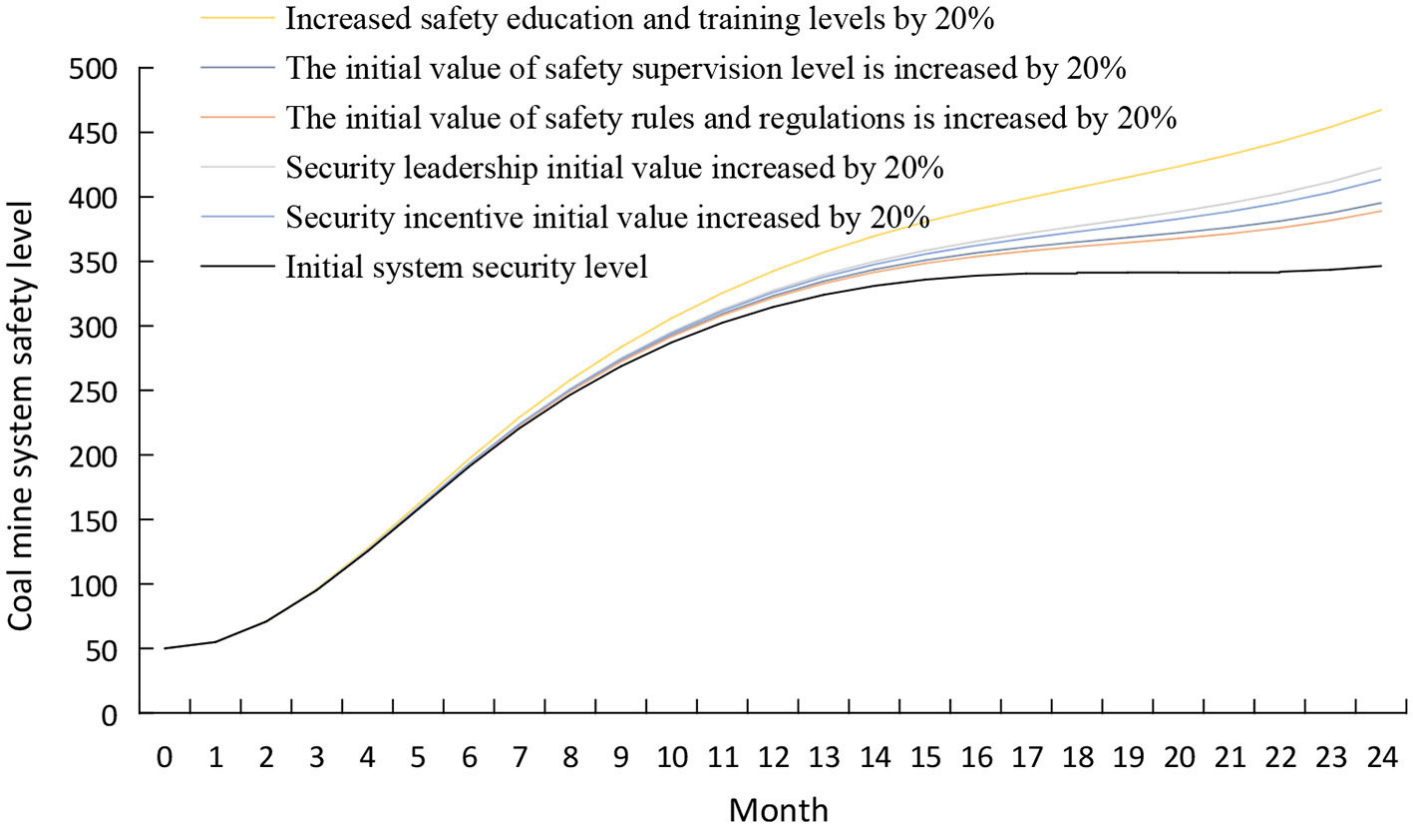
Simulation results of coal mine system safety after the intervention of the organizational management subsystem.

Under the condition of keeping other variables unchanged, using the control variable method increases the initial values of safety education and training, safety supervision, safety leadership, safety regulations, and safety incentives by 20%. The simulation results are shown in Figure 9. As seen in Figure 9, the safety state of the coal mine system has improved compared to the initial state after the intervention strategy. The impact of various factors on the safety of the coal mine system is from large to small: safety education and training, safety leadership, safety incentives, safety supervision level, and safety rules and regulations.

Strengthening safety education and training is the most economical and effective way for coal mining enterprises to improve safety performance (47). Increasing the initial value of safety education and training can improve managers' safety management levels and influence miners' subsystems. On the one hand, the increase in safety education and training level can improve miners' professional and technical levels and enhance their ability to deal with emergencies. On the other hand, the knowledge acquired through training is conducive to miners' understanding of the functions and intentions of intelligent systems so that miners can maintain an appropriate level of automation trust (48). Coal mining enterprises should establish a standardized and institutionalized safety education and training mechanism to ensure the strength and sustainability of safety education (49).

The safety leadership behavior of managers is critical to the safety management of coal mining enterprises (50). Leaders can control the age, specialties, and skills of members of the miner group through access management and optimize the group structure to reduce the occurrence of unsafe behaviors. In addition, leaders must correctly use the influence of authority, actively learn and master the legal standards and scientific methods of safety management, and seriously investigate the law of accidents to improve safety management (20).

Security incentives can effectively increase the safe behavior of miners. Managers can mobilize the enthusiasm and consciousness of employees for safety work through material and spiritual rewards, such as rewarding employees who truthfully reflect hidden dangers and risks; rewarding safety production teams, units, and individuals. When workers are encouraged, workers will proactively identify hazards and improve the safety atmosphere in the team (51).

Safety supervision is an essential factor influencing system safety. Managers can refer to BBS and Dupont STOP behavior management methods, formulate scientific and adequate supervision and assessment methods, arrange reasonable safety supervision cycles, and strictly implement safety supervision. Find and solve problems promptly in safe production (20), and create a good group safety atmosphere.

Safety rules and regulations can effectively regulate the behavior of miners. To improve the safety rules and regulations of coal mining enterprises, managers should conduct regular safety inspections, develop a miner's behavior management manual, and clarify guidelines for penalties for violations. In addition, managers need to clarify the criteria for analyzing and assessing miners' unsafe behaviors, establish individual miners' safety integrity files, and implement dynamic management of individual miners' safety behaviors (51).

#### The intervention in the group subsystem

**Figure 10:**
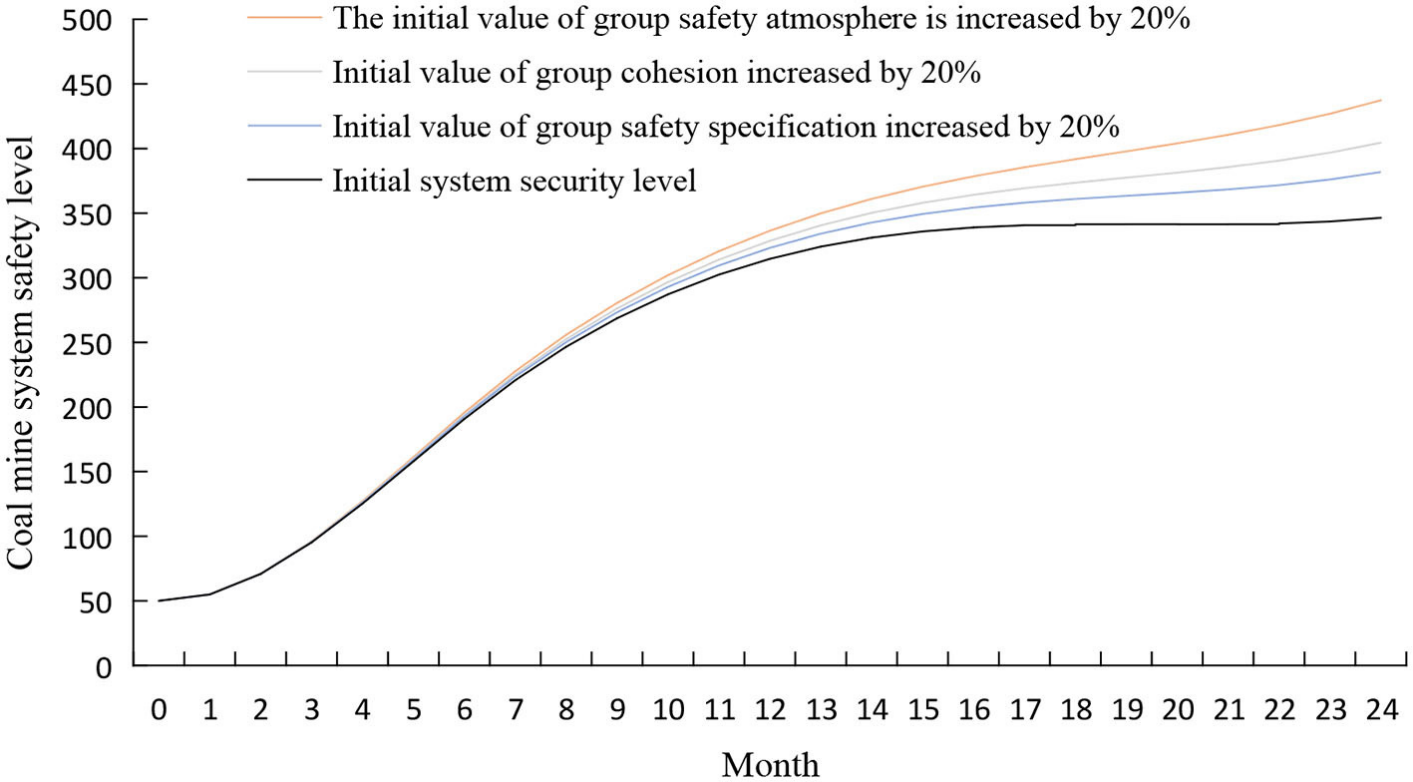
Simulation results of coal mine system safety after the intervention of group subsystem.

The dynamic complexity of coal mine hazards determines that organizational management cannot eliminate all safety risks, and employees must be encouraged to participate actively in safety management. The initial values of group safety climate, group cohesion, and group safety norm were increased by 20% using the control variable method while keeping other variables unchanged. The simulation results are shown in Figure 10. It can be seen that after adopting the intervention strategy, the effect of each factor on the safety level of the coal mine system is from large to small: group safety atmosphere, group cohesion, and group safety norms (52).

A good group safety atmosphere will reduce the unsafe behavior of miners by reducing the work pressure of miners, thereby leading to an increase in the safety level of the coal mine system (53). A good group safety atmosphere is mainly manifested in showing initiative, proposing changes to improve system security, and helping colleagues have sufficient resources to meet work needs. Team leaders can create a good group safety atmosphere by determining group safety goals and improving group safety standards.

Group cohesion positively influences the active participation of team members in group actions (54). It is necessary to encourage positive emotional interaction among team members and feedback on safety messages to improve team cohesion. The team leader should always pay attention to the members' status, promptly ease the miners' work pressure, and constructively resolve conflicts within the team (55, 56).

Ignoring safety regulations is the root cause of safety incidents (57). The first step to improve group safety norms is introducing standardized business concepts into group safety norms with teams as a unit. Before the team goes down the mine, carry out risk identification activities, regularly analyze unsafe behaviors in the group, improve workers' self-safety awareness, and give full play to the restraint and guiding role of safety norms in the group on miners.

#### The intervention in intelligent system subsystem

**Figure 11:**
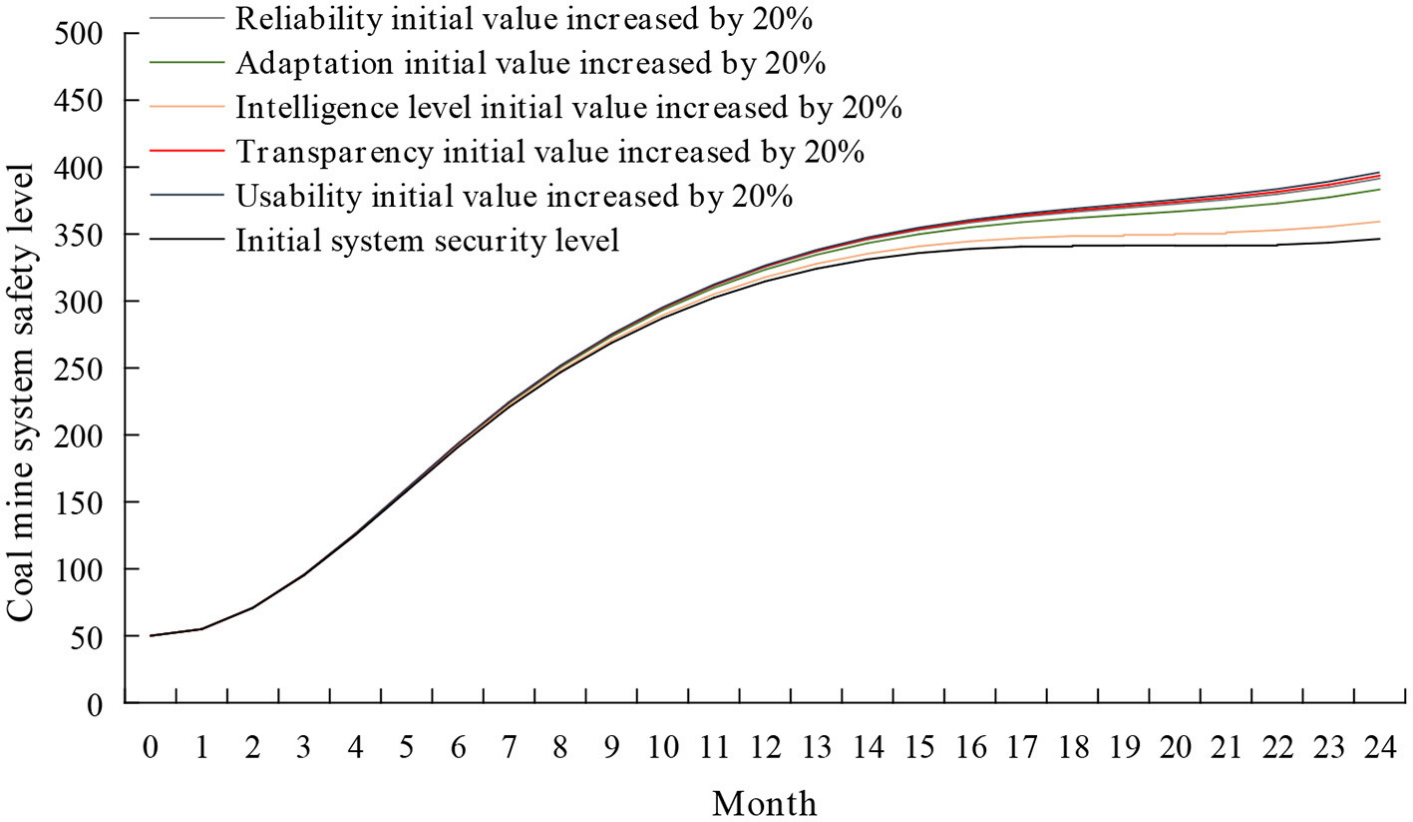
Simulation results of coal mine system safety after the intervention of the intelligent system subsystem.

The factor of an intelligent system is the most profound factor that influences the coal mine safety system, which will influence the decision-making of miners and the way of human-machine interaction. Using the control variable method while keeping other variables unchanged, the initial values of reliability, ease of use, transparency, intelligence level, and adaptability are increased by 20%. The simulation results are shown in Figure 11. It can be seen from Figure 11 that the effect of each factor on the safety level of the coal mine system is from large to small: ease of use, transparency, reliability, adaptability, and intelligence level.

The ease of use and transparency of intelligent systems directly impact miners' level of expertise. The higher the ease of use of the intelligent system, the simpler the operation method, and the fewer operation skills miners need to master, the easier it is to improve the professional technical level in a short period and reduce the unsafe behavior of miners. Higher system transparency can make it easier for miners to understand the system's operating principles through training and mastering the operating methods of the system, which can effectively improve the professional technical level of miners (58).

The reliability of intelligent systems influences miners' trust in the system and safety supervision (59). Intelligent systems with high reliability can increase miners' safety supervision of intelligent systems by setting appropriate human-machine task assignments, effectively reducing people's dependence on intelligent systems, and enabling miners to maintain an appropriate level of trust.

The adaptability of intelligent systems has a significant impact on the safe atmosphere of the group and the automation trust level of miners. High system adaptability can increase miners' safety supervision of machines and reduce people's excessive dependence on intelligent systems, effectively improving miners' trust level in intelligent systems and group safety atmosphere (29).

The intelligence of the intelligent system determines the degree to which miners participate in information acquisition, information analysis, decision-making, and decision-making in the human-computer interaction process. A higher level of intelligence can provide appropriate support for operators and reduce the workload of operators, thereby effectively balancing the work pressure of miners (60).

#### The intervention in the external environment subsystem

**Figure 12:**
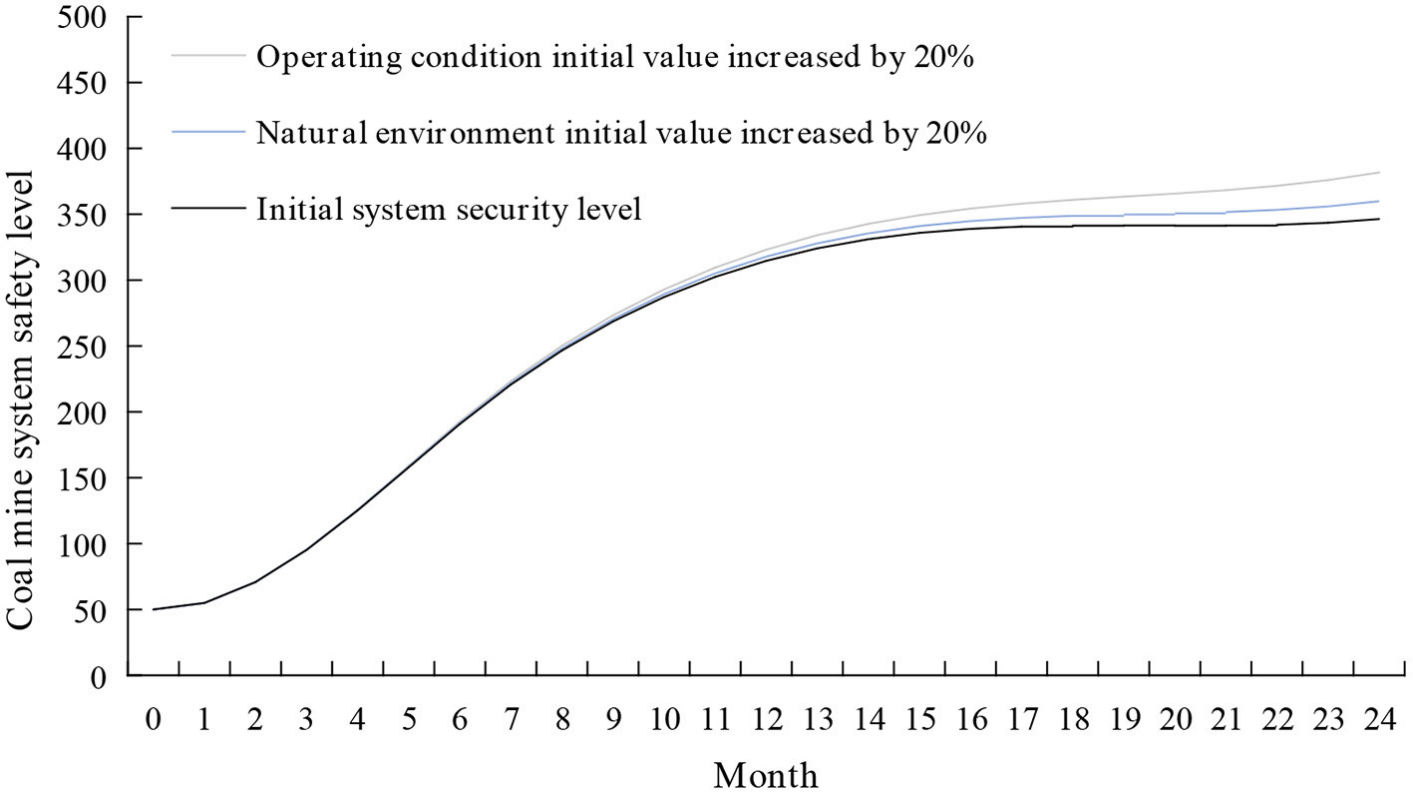
Simulation results of coal mine system safety after the intervention of external environment subsystem.

Using the control variable method while keeping other variables unchanged, the initial value of the natural environment and operating conditions is increased by 20%. The simulation results are shown in Figure 12. It can be seen from Figure 12 that the effect of each factor on the safety level of the coal mine system is from large to minor: operating conditions and natural environment.

Although operating conditions do not directly influence system security, environmental stimuli interact with the human-machine interaction process and influence miners' ability to complete tasks. A good environment is a prerequisite for miners' safe behavior. To improve the working environment of miners, on the one hand, it is necessary to actively introduce advanced tools and mechanical equipment to improve the safety level of coal mining equipment. On the other hand, it is also possible to increase the level of safe operation of miners by adjusting lighting, reducing noise, and generally improving working spaces (61).

## Conclusion

There is a complex interaction between the factors influencing the safety level of coal mine systems. This paper identifies 22 factors influencing coal mine system safety from five aspects: individual miners, organization and management, group, intelligent system, and environment. The ISM method was used to classify the 22 factors into five levels and to show the interaction relationships between the factors. The results of the ISM show that the individual miner factor is the most direct factor influencing system safety. The organizational management factor and group factor indirectly influence the coal mine system safety by influencing the individual miner, and the intelligent system is the most profound factor influencing the coal mine system safety. Through ANP calculation, the weight of each factor influencing the safety of the coal mine system is determined. The leading indicators are sorted by weight: organizational safety management, individual miners, groups, intelligent systems, and environment.

The mechanism of each influencing factor on the coal mine safety system was clarified. Based on the calculation results of ISM-ANP, the interaction relationship between the factors was clarified. We also constructed the system dynamics simulation model of the coal mine safety system and clarified the action mechanism of each factor of the safety system. It can be seen from the results that there are apparent differences in the influence of different subsystems on the security level of the system, which is determined by the interaction rules and interaction mechanisms between the systems. The organizational management subsystem has the most significant impact on system security among the five subsystems, followed by the miners' and group subsystems. The intelligent system and environmental subsystem have the most negligible impact on system security. The improvement of organizational management and group safety level can directly or indirectly influence miners' behavior, increase miners' safety behavior, and effectively promote the improvement of the safety level of the coal mine system. The positive effect of intelligent systems and environmental factors on the safety of coal mine systems is still being determined. However, an excellent operating environment and reliable equipment are the basis for ensuring system safety.

Develop intervention strategies to improve coal mine system safety. According to the simulation results of the model, after adopting different intervention strategies, the safety level of the coal mine system has increased in different ranges, indicating that the intervention strategy can effectively improve the system's safety level. The factors in each subsystem have different degrees of influence on the safety of the coal mine system, and each subsystem interacts, develops, and changes together. According to the simulation results, this paper proposes targeted intervention strategies from the specific operation level to optimize the coal mine safety management system and improve the system safety level.

## Limitations of the study

According to the ISM-ANP model results, each factor's action mechanism on the coal mine safety system and the importance degree of the factors are clarified. The simulation results of system dynamics have specific reference values for the safety management of the coal mine system. The proposed intervention strategy has guiding significance for the actual coal mine production. However, while getting the above research conclusions, this paper still has some things that could be improved. First, the index system of influencing factors of coal mine safety can be further improved. The indicators of the influencing factors of the coal mine safety system constructed in this paper are determined based on the analysis of the existing influencing factors at home and abroad combined with the actual coal mine safety production at the present stage. However, these studies are not comprehensive, and the follow-up research can be further supplemented and improved by other methods. Secondly, since the ISM-ANP model depends on the decision maker's experience, knowledge, and professional judgment, it is subjective to some extent. Therefore, the actual application of the model may result in different results due to the difference in the personal level of the decision maker. Therefore, more quantitative methods can be considered for subsequent research. Finally, the coal mine safety system is a considerable safety management system. The interaction rules between the model and factors constructed in this paper are simplified compared with the actual situation. There are some differences compared with the complexity of the existing safety system. In future research, the model will continue to be optimized to make the model closer to the actual production situation. The safety strategy explored in this paper will be applied to coal mine safety production.

## Data availability statement

The datasets presented in this article are not publicly available in order to protect the privacy of the respondents. Requests to access the datasets should be directed to KT, tian6039@126.com.

## Author contributions

XY and QX contributed to the study's design, data acquisition, data interpretation, model building, manuscript development, and revisions. KT contributed to data acquisition, data interpretation, manuscript development, and revisions. CL and JY contributed to data acquisition and manuscript revisions. All authors approved the final version of the submitted manuscript.

## Funding

This work was supported by the National Natural Science Foundation of China (71573086), the Graduate Student Innovation Project of North China University of Water Resources and Electric Power (YK-2021-119), Research on Deepening Industrial Open Development and Institutional Innovation in the Context of Henan Free Trade Zone Experimental Zone Version 2.0 A (2022-ZM-T06-01), Innovation Team of Network Public Opinion and Social Governance of North China University of Water Resources and Electric Power (01), Academic Degrees and Graduate Education Reform Project of Henan Province, China (2021SJGLX013Y), and Higher Education Reform and Practice Project of Henan Province, China (2021SJGLX159).

## Conflict of interest

The authors declare that the research was conducted in the absence of any commercial or financial relationships that could be construed as a potential conflict of interest.

## Publisher's note

All claims expressed in this article are solely those of the authors and do not necessarily represent those of their affiliated organizations, or those of the publisher, the editors and the reviewers. Any product that may be evaluated in this article, or claim that may be made by its manufacturer, is not guaranteed or endorsed by the publisher.
